# Changes in Temperature Have Opposing Effects on Current Amplitude in α7 and α4β2 Nicotinic Acetylcholine Receptors

**DOI:** 10.1371/journal.pone.0032073

**Published:** 2012-02-16

**Authors:** Marie Jindrichova, Stuart J. Lansdell, Neil S. Millar

**Affiliations:** 1 Department of Neuroscience, Physiology and Pharmacology, University College London, London, United Kingdom; 2 Institute of Physiology of the Academy of Sciences of the Czech Republic, Prague, Czech Republic; University of Bristol, United Kingdom

## Abstract

We have examined the effect of temperature on the electrophysiological properties of three neuronal nicotinic acetylcholine receptor (nAChR) subtypes: the rapidly desensitizing homomeric α7 nAChR, the more slowly desensitizing heteromeric α4β2 nAChR and on α7 nAChRs containing a transmembrane mutation (L247T) that results in dramatically reduced desensitization. In all cases, the functional properties of receptors expressed in *Xenopus* oocytes at room temperature (RT; 21°C) were compared to those recorded at either physiological temperature (37°C) or at lower temperature (4°C). Alterations in temperature had dramatically differing effects on the amplitude of whole-cell responses detected with these three nAChR subtypes. Compared to responses at RT, the amplitude of agonist-evoked responses with α4β2 nAChRs was increased at high temperature (125±9%, *n* = 6, *P*<0.01) and reduced at low temperature (47±5%, *n* = 6, *P*<0.01), whereas the amplitude of α7 responses was reduced at high temperature (27±7%, *n* = 11, *P*<0.001) and increased at low temperatures (224±16%, *n* = 10, *P*<0.001). In contrast to the effects of temperature on α4β2 and wild type α7 nAChRs, the amplitude of α7 nAChRs containing the L247T mutation was unaffected by changes in temperature. In addition, changes in temperature had little or no effect on current amplitude when α7 nAChRs were activated by the largely non-desensitizing allosteric agonist 4BP-TQS. Despite these differing effects of temperature on the amplitude of agonist-evoked responses in different nAChRs, changes in temperature had a consistent effect on the rate of receptor desensitization on all subtypes examined. In all cases, higher temperature resulted in increased rates of desensitization. Thus, it appears that the differing effects of temperature on the amplitudes of whole-cell responses cannot be explained by temperature-induced changes in receptor desensitization rates.

## Introduction

Nicotinic acetylcholine receptors (nAChRs) are members of the Cys-loop family of ligand-gated ion channels which also includes receptors for 5-hydroxytryptamine (5-HT), γ-aminobutyric acid (GABA) and glycine [1]. In common with other Cys-loop receptors, nAChRs are oligomeric transmembrane proteins in which five subunits co-assemble to form a central ion-channel pore [2]. In addition, subunits of Cys-loop receptors share a common transmembrane topology, containing an extracellular N-terminal region and four α-helical transmembrane domains [1].

Seventeen nAChRs subunits (α1–α10, β1–β4, γ, δ and ε) have been identified in vertebrate species and can co-assemble into a large number of nAChR subtypes with considerable diversity in subunit composition [3]. In most cases, nAChRs are heteromeric complexes (containing two, three or four different subunit subtypes) but some subunits, such as α7, are capable of forming homomeric receptors (containing five copies of a single subunit). Within the mammalian brain, two nAChR subtypes (heteromeric α4β2 nAChRs and homomeric α7 nAChRs) have attracted particular attention as targets for pharmaceutical drug discovery. Homomeric α7 nAChRs have high calcium permeability and very rapid desensitization. They have been identified as potential drug targets in treatment of disorders such as Alzheimer's disease and schizophrenia [4], [5], [6]. Heteromeric α4β2 nAChRs have lower calcium permeability and display less agonist-induced desensitization. Receptors containing α4 and β2 subunits mediate the effects of nicotine associated with tobacco smoking and are the site of action of drugs used to assist with smoking cessation [7]. In addition, α4β2 nAChRs are targets for drug discovery in areas such as cognition, attention and pain [4], [5], [6].

A variety of experimental approaches have confirmed that conventional orthosteric agonists such as acetylcholine bind at an extracellular site located at the interface of two subunits [8], [9]. More recently, studies with α7 nAChRs have demonstrated that nAChRs can also be activated by agonists binding to an allosteric site located in the transmembrane region [10], a site that has previously been proposed as the binding site for a range of allosteric modulators of α7 nAChRs [11], [12]. Whereas activation of α7 nAChRs by acetylcholine results in rapid desensitization [13], activation by allosteric agonists such as 4BP-TQS results in very low levels of desensitization [10], consistent with these two agonists having different mechanisms of action. Previous studies have also demonstrated that the rapid rate of desensitization observed when α7 nAChRs is activated by orthosteric agonists such as acetylcholine can be reduced dramatically by the introduction of a single point mutation (L247T) located within the second transmembrane domain [14].

A particular advantage of studies conducted with recombinant nAChRs in artificial expression systems is the ability to examine the properties of receptors with defined subunit composition, as well as the ability to examine the effects of alterations in amino acid composition by means of techniques such as site-directed mutagenesis [15]. Typically, electrophysiological studies of recombinant nAChRs are conducted at room temperature, as are studies of native receptors from isolated cell and tissue preparations. In the present study we have examined the influence of conducting electrophysiological recordings at temperatures above and below room temperature (37°C and 4°C). By means of expression studies in *Xenopus* oocytes, we have examined heteromeric α4β2 and homomeric α7 nAChRs. In addition, we have examined the effect of changes in temperature on responses evoked by both orthosteric and allosteric agonists, as well as on α7 nAChRs containing a mutation that slows the rate of desensitization caused by orthosteric agonists such as acetylcholine. Changes in temperature resulted in changes in the magnitude of agonist-evoked responses. However, opposing effects were observed on different nAChR subtypes. Changes in temperature were also associated with changes in rates of receptor desensitization, but this does not appear to explain the differences observed in current amplitudes at different temperatures.

## Results

### Effect of temperature on current amplitude in α7 nAChRs

**Figure 1:**
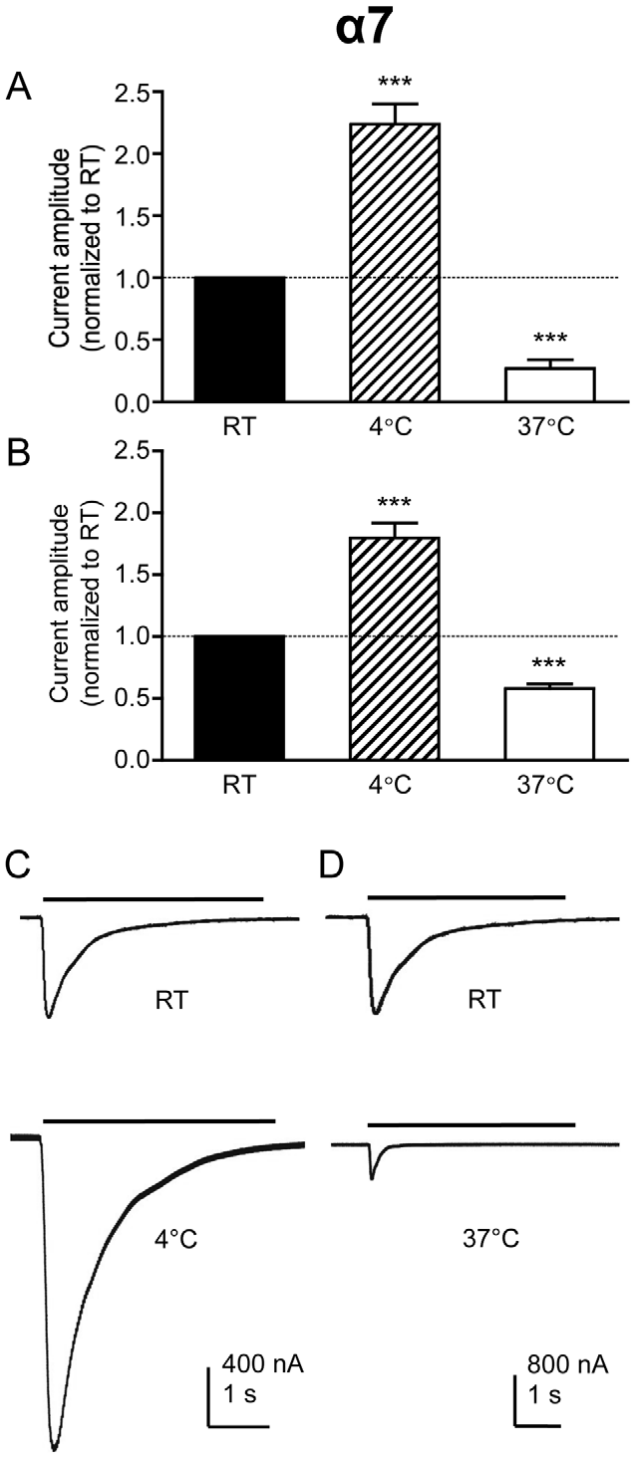
Electrophysiological characterization of α7 nAChRs expressed in *Xenopus* oocytes in response to acetylcholine. Bar charts illustrate responses (mean ± SEM) from α7 nAChRs expressed in *Xenopus* oocytes in response to a maximal (3 mM) and *EC*_50_ (100 µM) concentration of acetylcholine (A and B, respectively) at room temperature (RT; 21°C), higher temperature (37°C) and lower temperature (4°C). Data are means of 7–11 responses, each from a different oocyte, in which responses obtained at either 4°C or 37°C are normalized to responses obtained from the same oocyte at RT. C) Representative traces illustrating responses obtained at RT (upper trace) and 4°C (lower trace) from a single oocyte. D) Representative traces illustrating responses obtained at RT (upper trace) and 37°C (lower trace) from a single oocyte.

Expression of the human α7 nAChR in *Xenopus* oocytes was examined by two-electrode voltage-clamp recording. When acetylcholine-evoked responses were recorded at room temperature (RT; 21°C), rapidly desensitizing currents were observed (Fig. 1), typical of α7 nAChRs [13]. When responses were recorded from the same oocytes at physiological temperature (37°C), agonist-evoked current amplitudes were significantly smaller (*P*<0.001; Fig. 1, Table 1). This was the case, irrespective of whether a maximal concentration (3 mM; Fig. 1A) or an *EC*_50_ concentration (100 µM; Fig. 1B) of acetylcholine or was used. Conversely, when responses were recorded at lower temperature (4°C), currents were significantly larger (*P*<0.001; Fig. 1, Table 1). Again, this was the case, irrespective of whether a maximal concentration or an *EC*_50_ concentration of acetylcholine or was used (Fig. 1, Table 1).

**Table 1:** Amplitude and desensitization of nAChR responses examined at different temperatures.

Receptor	Current amplitude normalized to RT (%)			Desensitization (either s† or %††)		
	RT	4°C	37°C	RT	4°C	37°C
α7 (3 mM ACh)	100	224±16 (*n* = 10) ***	27±7 (*n* = 11) ***	0.39±0.04 (*n* = 21)†	0.62±0.07 (*n* = 10)† **	0.20±0.04 (*n* = 11)† **
α7 (100 µM ACh)	100	180±12 (*n* = 7) ***	58±4 (*n* = 7) ***	0.93±0.07 (*n* = 12)†	1.0±0.12 (*n* = 7)†	0.83±0.15 (*n* = 6)†
α4β2 (Ca^2+^ saline)	100	47±5 (*n* = 6) **	125±9 (*n* = 6) **	12.3±2.36 (*n* = 13)††	1.79±1.11 (*n* = 6)†† **	23.3±4.08 (*n* = 6)†† *
α4β2 (Ba^2+^ saline)	100	50±5 (*n* = 9) ***	127±6 (*n* = 6) **	9.69±2.40 (*n* = 15)††	3.55±1.80 (*n* = 9)†† *	14.9±1.26 (*n* = 6)†† ***
α7^L247T^ (30 µM ACh)	100	99±3 (*n* = 9)	106±5 (*n* = 8)	3.86±0.70 (*n* = 17)††	2.17±0.61 (*n* = 10)††	7.97±1.40 (*n* = 7)†† ***
α7^L247T^ (0.4 µM ACh)	100	102±9 (*n* = 5)	100±8 (*n* = 6)	3.13±1.25 (*n* = 10)††	1.04±0.73 (*n* = 5)††	4.99±2.87 (*n* = 5)††
α7 (10 µM 4BP-TQS)	100	118±7 (*n* = 19)	80±5 (*n* = 22) **	1.66±0.49 (*n* = 26)††	1.21±0.39 (*n* = 12)††	9.09±0.79 (*n* = 16)†† ***

†, †† Data for desensitization of all receptor and agonist combinations (with the exception of wild-type α7, activated by ACh) are expressed as the percentage of decay from the peak response in 5 seconds. Due to the rapid rate of desensitization for wild-type α7 activated by ACh, these values are expressed as the time required for the response to decay to 50% of the peak response.

Data are means ± SEM. Significant differences to responses recorded at RT are indicated (* = *P*<0.05, ** = *P*<0.01, *** = *P*<0.001).

### Effect of temperature on current amplitude in α4β2 nAChRs

**Figure 2:**
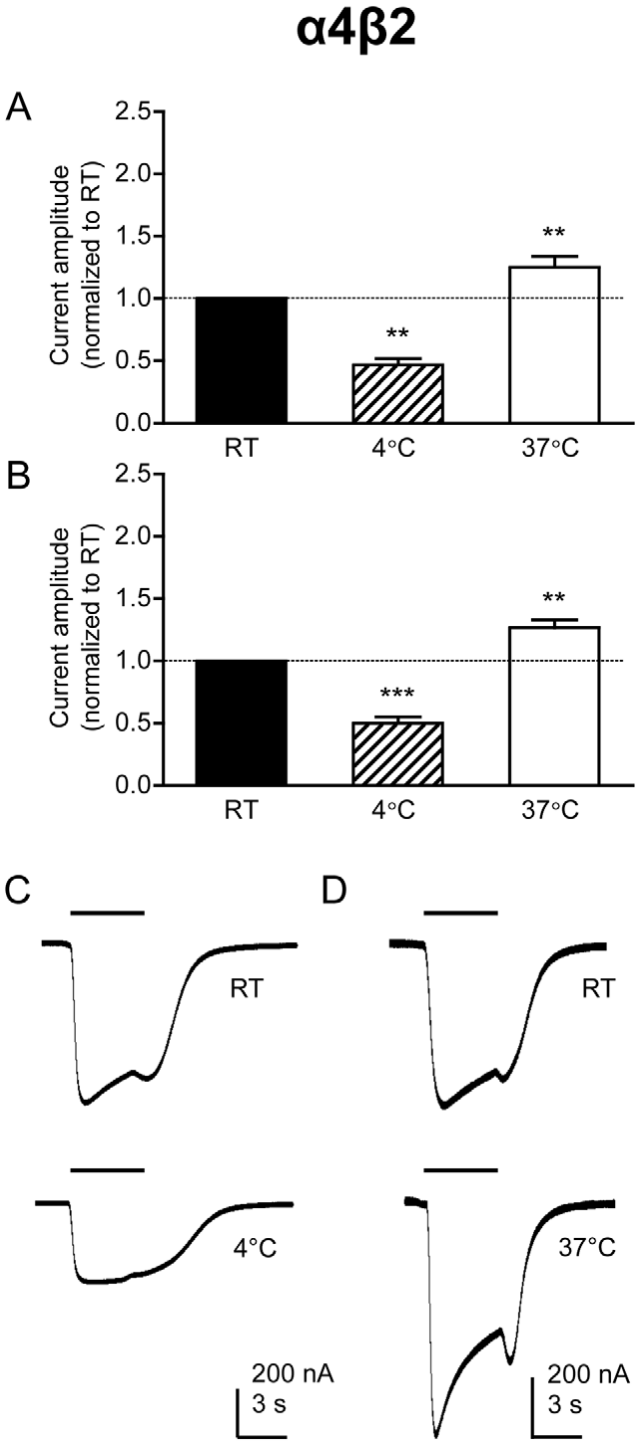
Electrophysiological characterization of α4β2 nAChRs expressed in *Xenopus* oocytes in response to acetylcholine. Bar charts illustrate responses (mean ± SEM) from α4β2 nAChRs expressed in *Xenopus* oocytes in response to a maximal (1 mM) concentration of acetylcholine in either calcium-contanining (A) or barium-containing Ringer solution (B) at room temperature (RT; 21°C), higher temperature (37°C) and lower temperature (4°C). Data are means of 6–9 responses, each from a different oocyte, in which responses obtained at either 4°C or 37°C are normalized to responses obtained from the same oocyte at RT. C) Representative traces illustrating responses obtained at RT (upper trace) and 4°C (lower trace) from a single oocyte. D) Representative traces illustrating responses obtained at RT (upper trace) and 37°C (lower trace) from a single oocyte. Representative traces are from calcium-containing saline but similar responses were obtained with barium-containing saline.

A similar series of experiments was performed with the human α4β2 nAChR subtype, which displays lower levels of desensitization than homomeric α7 nAChRs (Fig. 2). With α4β2 nAChRs, the effect of temperature on current amplitude was the opposite of that observed with α7 nAChRs. When responses were recorded from oocytes at 37°C, agonist-evoked current amplitudes were significantly larger than at RT (*P* = 0.002; Fig. 2, Table 1). Conversely, when responses were recorded at 4°C, currents were significantly smaller than at RT (*P* = 0.002; Fig. 2, Table 1).

Whereas oocyte recordings with α4β2 nAChRs were conducted in calcium-containing saline, recordings with α7 nAChRs were conducted in barium-containing (calcium-free) saline. A calcium-free saline is typically used with α7 nAChRs to minimize the possibility of calcium-activated chloride channels (due to the higher calcium permeability of α7 nAChRs). In order to examine whether differences in saline composition might explain differences in the effect of temperature on current amplitudes, experiments with α4β2 nAChRs were repeated in calcium-free saline. The effect of temperature on current amplitude with α4β2 nAChRs was consistent between the two saline solutions (Fig. 2, Table 1). In addition, further experiments were performed with α7 nAChRs to examine the effects of temperature on responses recorded in calcium-containing saline, rather than in barium-containing saline. The effect of both high (37°C) and low (4°C) temperature on current amplitude of α7 nAChRs was not significantly different to that observed in barium-containing saline (n = 5).

### Effect of temperature on current amplitude in α7^L247T^ nAChRs

**Figure 3:**
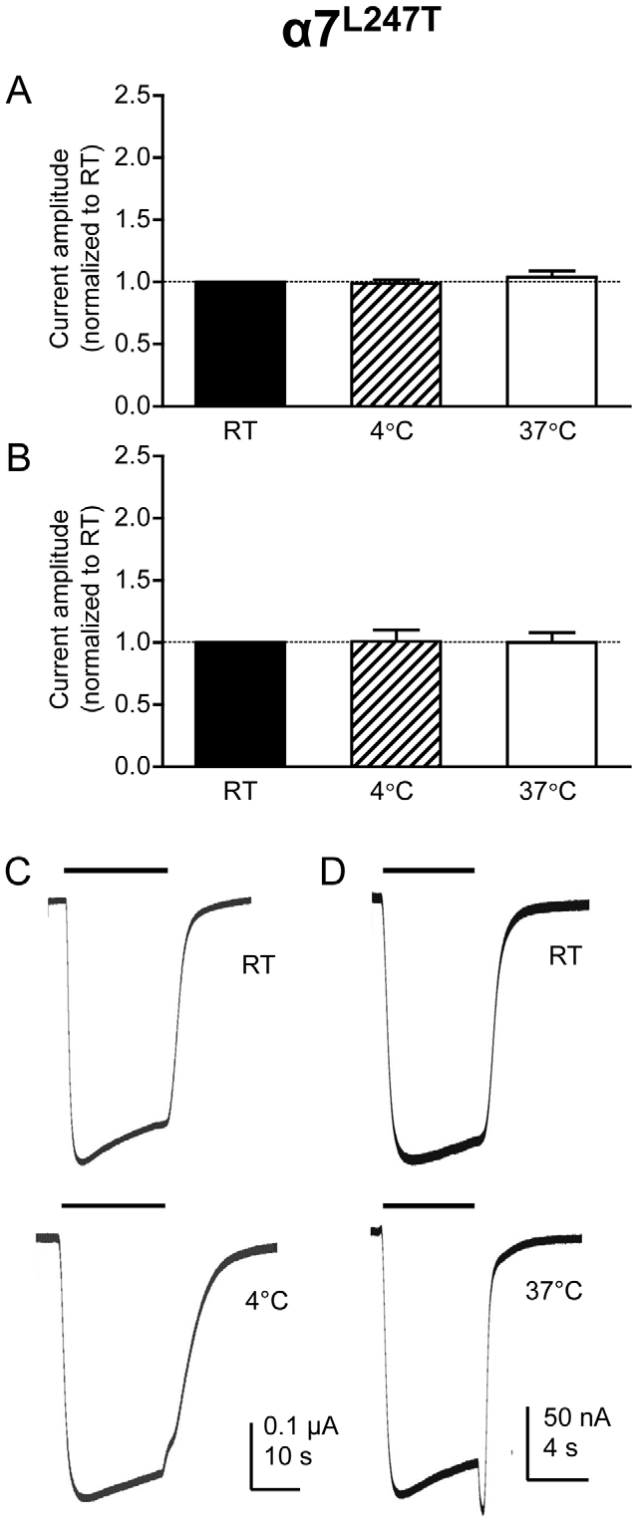
Electrophysiological characterization of α7^L247T^ nAChRs expressed in *Xenopus* oocytes in response to acetylcholine. Bar charts illustrate responses (mean ± SEM) from α7^L247T^ nAChRs expressed in *Xenopus* oocytes in response to a maximal (30 µM) and *EC*_50_ (0.4 µM) concentration of acetylcholine (A and B, respectively) at room temperature (RT; 21°C), higher temperature (37°C) and lower temperature (4°C). Data are means of 5–9 responses, each from a different oocyte, in which responses obtained at either 4°C or 37°C are normalized to responses obtained from the same oocyte at RT. C) Representative traces illustrating responses obtained at RT (upper trace) and 4°C (lower trace) from a single oocyte. D) Representative traces illustrating responses obtained at RT (upper trace) and 37°C (lower trace) from a single oocyte.

A further series of experiments was performed with the human α7 nAChR containing a single point mutation (L247T) in the second transmembrane domain. As has been described previously [14], one consequence of this mutation is a reduction in the extent of receptor desensitization (Fig. 3). In contrast to the situation with the wild-type α7 nAChR (Fig. 1), no significant difference was observed in the effect of temperature on the amplitude of responses evoked by acetylcholine on α7 nAChRs containing the L247T mutation (Fig. 3). This was the case, irrespective of whether a maximal concentration (30 µM) or an *EC*_50_ concentration (0.4 µM) of acetylcholine or was used (Fig. 3, Table 1).

### Effect of temperature on α7 nAChRs activated by 4BP-TQS

**Figure 4:**
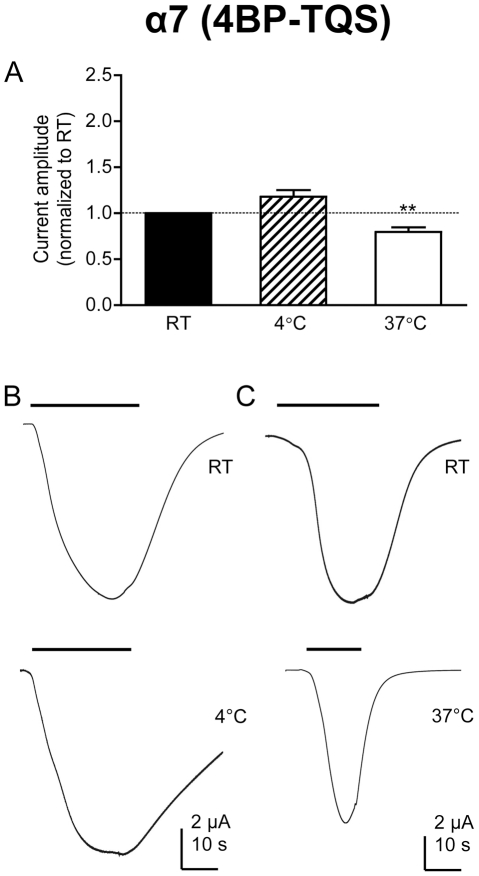
Electrophysiological characterization of α7 nAChRs expressed in *Xenopus* oocytes in response to 4BP-TQS. A) A bar chart illustrates responses (mean ± SEM) from α7 nAChRs expressed in *Xenopus* oocytes in response to a maximal (10 µM) concentration of the allosteric agonist 4BP-TQS at room temperature (RT; 21°C), higher temperature (37°C) and lower temperature (4°C). Data are means of 5–22 responses, each from a different oocyte, in which responses obtained at either 4°C or 37°C are normalized to responses obtained from the same oocyte at RT. B) Representative traces illustrating responses obtained at RT (upper trace) and 4°C (lower trace) from a single oocyte. C) Representative traces illustrating responses obtained at RT (upper trace) and 37°C (lower trace) from a single oocyte.

Whereas activation of wild-type α7 nAChRs by acetylcholine results in rapidly desensitizing responses, activation of α7 nAChRs by the allosteric agonist 4BP-TQS has a much slower onset and causes much less desensitization [10]. The effect of changes in temperature was examined on α7 nAChRs after activation by 4BP-TQS (Fig. 4). As reported previously [10], slowly activating responses were observed with minimal levels of desensitization. In contrast to the marked effect of temperature changes on the amplitude of currents evoked by acetylcholine on α7 nAChRs (Fig. 1), temperature changes had little or no effect on responses to 4BP-TQS (Fig. 4). Lower temperature had no significant effect and higher temperature caused a relatively small but statistically significant (*P* = 0.002), reduction in current amplitude.

One possible explanation for these results is that the relatively small effect of high temperature on current amplitude in α7 nAChRs activated by 4BP-TQS might be due to instability of the compound at 37°C. To examine this possibility, control experiments were performed with solutions of 4BP-TQS that had been stored at 37°C for three hours and then cooled to room temperature. No differences in agonist responses were observed, when compared with freshly prepared solutions (data not shown).

### Effect of temperature on receptor desensitization and deactivation

**Figure 5:**
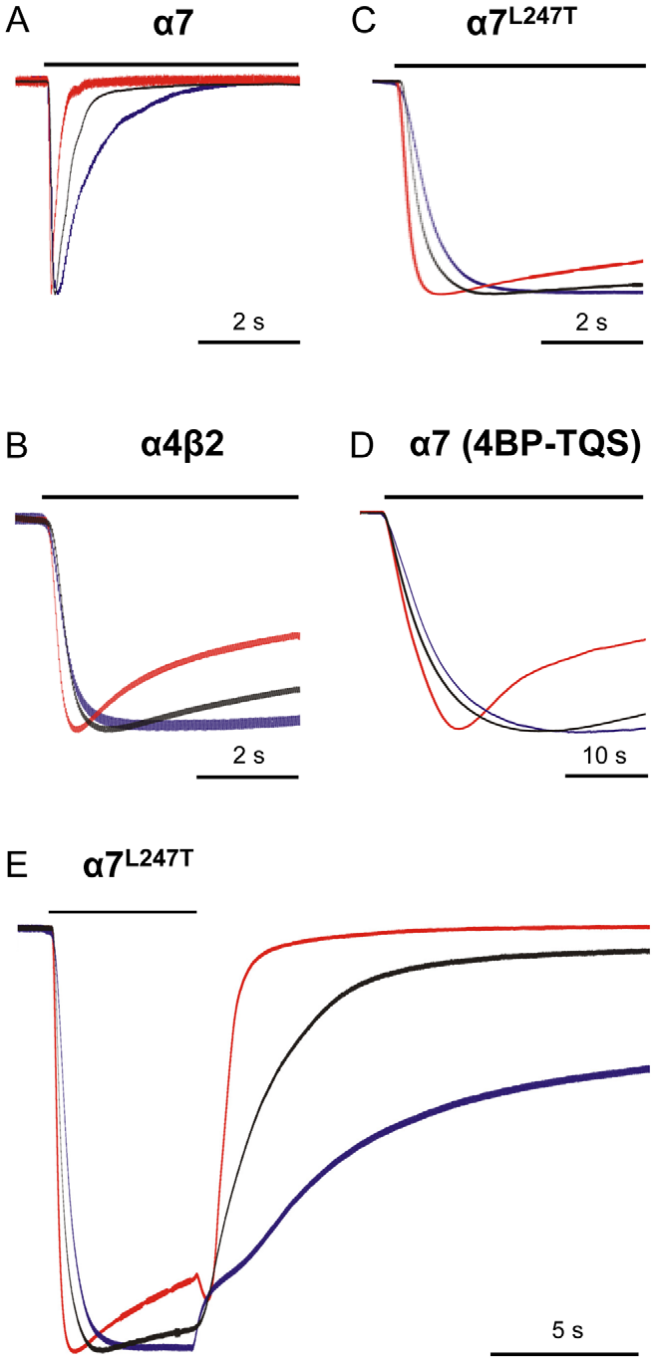
Electrophysiological characterization of nAChRs expressed in *Xenopus* oocytes examined at different temperatures. Representative traces are shown illustrating responses obtained at RT (black), 4°C (blue) and 37°C (red). Current traces obtained at each temperature have been normalized to the same peak response. In each case, the response showing the fastest rate of desensitization was observed at 37°C and the slowest rate of desensitization was observed at 4°C. Responses are from α7 nAChRs with 3 mM acetylcholine (A), α4β2 nAChRs with 1 mM acetylcholine in calcium-containing saline (B), α7^L247T^ nAChRs with 30 µM acetylcholine (C) and α7 nAChRs with 10 µM 4BP-TQS (D). Rates of receptor deactivation after removal of agonist were also influenced in a consistent manner by changes in temperature (faster at 37°C and slower at 4°C). Representative traces from α7^L247T^ nAChRs with 30 µM acetylcholine are illustrated (E) and are typical of results from all receptor/agonist combinations studied (see Tables 1 and 2 for details).

In addition to examining changes in current amplitude, the effect of altering temperature was examined on the rate of receptor desensitization (Fig. 5). Despite both high and low temperatures having opposing effects on current amplitudes on α4β2 and α7 nAChRs, changes in temperature had a consistent effect on the rate of receptor desensitization for α4β2 and α7 nAChRs. For both receptor subtypes (α4β2 and α7), an increase in the rate of desensitization was observed at 37°C and a decrease in the rate of desensitization at 4°C (Fig. 5). For both α7^L247T^ nAChRs and for wild-type α7 nAChRs activated by 4BP-TQS, despite changes in temperature having little or no effect on current amplitude (Figs. 3 and 4), an increase in the rate of receptor desensitization was observed at 37°C, consistent with the effects seen with α4β2 and wild-type α7 nAChRs (Fig. 5). There was also evidence of a reduction in the rate of desensitization at 4°C for both α7^L247T^ nAChRs and for wild-type α7 nAChRs activated by 4BP-TQS, consistent with the effects seen with α4β2 and wild-type α7 nAChRs, but this was not statistically significant (Fig. 5, Table 2). The influence of changes in temperature was also examined on the rate of receptor deactivation after removal of agonist (Fig. 5E). It was not possible to measure this parameter for of α7 nAChRs activated by acetylcholine, due to the very rapid desensitization but, in all other cases, changes in temperature had a consistent effect (Table 2), with an increased rate of deactivation observed at 37°C and a reduced rate at 4°C.

**Table 2:** Rise time and deactivation of nAChR responses examined at different temperatures.

Receptor	Rise time (s)			Deactivation (s)†		
	RT	4°C	37°C	RT	4°C	37°C
α7 (3 mM ACh)	0.22±0.03 (*n* = 21)	0.26±0.03 (*n* = 10)	0.14±0.03 (*n* = 11) *	-	-	-
α7 (100 µM ACh)	0.65±0.03 (*n* = 13)	0.88±0.08 (*n* = 7) *	0.4±0.03 (*n* = 6) ***	-	-	-
α4β2 (Ca^2+^ saline)	1.57±0.20 (*n* = 13)	2.39±0.30 (*n* = 6)	1.04±0.18 (*n* = 6)	2.97±0.17 (*n* = 12)	3.85±0.26 (*n* = 7) ***	1.91±0.35 (*n* = 5) ***
α4β2 (Ba^2+^ saline)	1.92±0.30 (*n* = 15)	2.11±0.32 (*n* = 9)	1.50±0.33 (*n* = 6)	3.19±0.29 (*n* = 13)	3.67±0.49 (*n* = 6) **	2.35±0.34 (*n* = 7) ***
α7^L247T^ (30 µM ACh)	3.16±0.27 (*n* = 14)	4.12±0.46 (*n* = 8)	2.02±0.30 (*n* = 6) *	1.62±0.19 (*n* = 14)	2.80±0.36 (*n* = 8) ***	0.72±0.27 (*n* = 6) ***
α7^L247T^ (0.4 µM ACh)	3.66±0.63 (*n* = 10)	4.38±0.42 (*n* = 5)	3.21±0.72 (*n* = 5)	1.34±0.13 (*n* = 7)	2.90±0.15 (*n* = 3) *	0.81±0.16 (*n* = 4) *
α7 (10 µM 4BP-TQS)	33.5±3.68 (*n* = 24)	36.1±5.25 (*n* = 8)	15.5±1.69 (*n* = 15) ***	27.3±4.94 (*n* = 21)	131.9±34.1 (*n* = 13) ***	8.13±2.0 (*n* = 9) ***

† Data for deactivation are expressed as the time required for the response to decay to 50% of the response after termination of agonist application. This parameter could not be determined for wild-type α7 activated by ACh, due to the rapid rate of agonist-induced desensitization.

Data are means ± SEM. Significant differences to responses recorded at RT are indicated (* = *P*<0.05, ** = *P*<0.01, *** = *P*<0.001).

### Effects of temperature are reversible

For all experiments, the effects of raising or lowering temperature on agonist responses were normalized to responses recorded at RT on the same oocyte. In addition, after responses had been recorded at a temperature above or below RT, further responses were measured on the same oocyte at RT. In all cases, changes in current amplitude or in the rate of receptor desensitization were found to be reversible (data not shown).

## Discussion

It has been known for several decades that changes in temperature can influence the properties of nAChRs. For example, a series of studies conducted in the early 1970s demonstrated that end-plate currents recorded at the frog neuromuscular junction are influenced by temperature [16], [17], [18], [19], [20]. Typically, the effects observed include increased rates of decay of end-plate currents with increased temperature. This is consistent with the increase in the rate of receptor desensitization that we have observed with both α4β2 and α7 nAChRs. Similarly, previous studies conducted with rat diaphragm preparations reported similar effects of temperature on rates of decay of end-plate currents [21]. Studies of native neuronal nAChRs expressed in cultured PC12 cells also demonstrated that rates of recovery from desensitization were faster at higher temperatures [22]. Thus, it seems that changes in temperature appear to have a consistent effect on the rate of agonist-induced desensitization and the decay of end-plate potentials.

An increase in current amplitude with increased temperature, similar to the effect we have observed with α4β2 nAChRs, has been reported recently for recombinant P2X_3_ receptors [23]. However, in contrast to the findings reported here, the development of desensitization in P2X_3_ receptors has been reported to be independent of temperature, at least between 25°C and 40°C [23]. A similar increase in current amplitude with increased temperature has also been reported for NMDA-type glutamate receptors [24]. Conversely, an increase in current amplitude in response to decreased temperature, similar to the effect that we observe with α7 nAChRs, has been reported with an acetylcholine-gated chloride channel from the parietal ganglion of the pond snail *Lymnaea* [25]. Thus, there are precedents for changes in temperature having opposing effects on the amplitude of ion channel currents, albeit from different classes of ligand-gated ion channels. In addition, we have recently become aware of a paper, published after the submission of this manuscript, examining the influence of higher temperature on α7 nAChRs [26]. Higher temperature resulted in reduced responses to acetylcholine when co-applied with the positive allosteric modulator PNU-120597, consistent with our results obtained with either acetylcholine or 4BP-TQS applied alone.

We have found that introduction of a single point mutation (L247T) in the α7 nAChR completely abolishes the effect of temperature on current amplitude, whilst retaining an effect of temperature on the rate of desensitization of the macroscopic response. This observation adds to the many effects that have been reported for the L247T mutation in α7 nAChRs. These include an increase in agonist potency, reduction in receptor desensitization, increased spontaneous openings, the conversion of competitive antagonists into agonists and the conversion of allosteric potentiators into allosteric agonists [10], [14], [27], [28]. As has been discussed previously [10], the non-desensitizing responses observed when α7 is activated by 4BP-TQS resembles that of acetylcholine responses on α7^L247T^ nAChRs. Changes in temperature had a greatly reduced effect on the amplitude of α7 responses in response to 4BP-TQS compared to responses to acetylcholine. Indeed, no significant effect was observed when responses at 4°C were compared to those at RT (Fig. 4). This is consistent with previous evidence [10] indicating that acetylcholine and 4BP-TQS cause activation of α7 nAChRs through different mechanisms of action.

It has been reported previously that exposure of some nAChRs to low temperature for several hours can facilitate more efficient protein folding and assembly. This was first demonstrated for the muscle-type nAChR from the marine ray *Torpedo*, expressed in cultured mammalian cell lines [29] and can be explained by the fact that proteins in cold water fish have not evolved to fold efficiently at 37°C. Similar effects have been reported for insect nAChRs expressed in cultured cell lines [30], [31], [32]. In fact, even mammalian nAChRs have been reported to fold and assemble more efficiently at lower temperature [33], [34]. However, in contrast to these long-term effects occurring over several hours, it is unlikely that increased efficiency of subunit folding and assembly explains the effects reported here. The effects of temperature on current amplitude examine in this study are both very rapid (occurring within seconds) and are reversible, suggesting that the effects observed are a consequence of a change in the thermodynamic properties of already assembled cell-surface receptors, rather than a change in the efficiency of receptor folding and assembly. In contrast to the experiments examining agonist responses at physiological temperature, the rationale for examining responses of human nAChRs at 4°C is probably less obvious. Although responses measured at 4°C do not have direct physiological relevance for human receptors, the fact that lowering temperature to 4°C has opposing effects on α4β2 and α7 nAChRs provides evidence that these two nAChR subtypes have differing biophysical properties.

It is unclear why changes in temperature should have opposing effects on current amplitude in two closely related ion channels (α4β2 and α7 nAChRs) or why these effects should be largely or completely abolished by either a single point mutation or by activation by an allosteric agonist, rather than by the conventional orthosteric agonist, acetylcholine. It appears, however, that these effects are not a consequence of changes in the rate of receptor desensitization observed during macroscopic oocyte responses, since changes in temperature were found to have a broadly consistent effect on this parameter. In addition, changes in temperature had a consistent effect on the rate of receptor deactivation after removal of agonist (faster at 37°C and slower at 4°C), presumably reflecting changes in the off-rate of agonists from their binding sites. It is possible that receptors can adopt multiple open or desensitized states, and that entry into these various states occurs at different rates and can be affected differently by phenomena such as changes in temperature, mutagenesis or by allosteric modulators. This idea is consistent with models that have been advanced to explain mechanisms of allosteric modulation of nAChRs [35].

It is possible that the changes in current amplitude that we have observed with α4β2 and α7 nAChRs may be a consequence of temperature-induced changes in single-channel conductance. Indeed, it has been reported that the conductance of muscle-type nAChRs increases with increasing temperature [36], [37], [38], [39]. As has been discussed previously in connection with the effects of temperature on functional properties of acetylcholine-gated chloride channels from the pond snail *Lymnaea* [25], alternative possibilities for the effects that we have observed include temperature-dependent changes in single-channel kinetics and/or changes in the affinity of agonist binding. Recently, evidence has been obtained indicating that the gating rate constants of muscle nAChRs are altered by temperature [40]. Further work will be required to establish the precise mechanism of action of the temperature dependent effects that we have observed on the amplitudes of whole-cell responses in different subtypes of neuronal nAChRs. We can conclude, however, that changes in temperature can have opposing effects on the amplitude of nAChR whole-cell responses, whilst having a similar effect on the rate of desensitization of the macroscopic agonist-evoked response.

## Materials and Methods

### Plasmids, site-directed mutagenesis and cRNA synthesis

Plasmid pSP64GL constructs containing human α4, α7 and β2 cDNA have been described previously [41] as has a plasmid containing the α7 cDNA with the L247T mutation [10]. All pSP64GL plasmids were linearized with *Bam*HI and purified with QIAquick PCR purification kit (Qiagen). *In vitro* synthesis of cRNA was performed using the SP6 mMessage mMachine SP6 kit (Ambion).

### Two-electrode voltage-clamp recording in *Xenopus* oocytes

Adult female *Xenopus laevis* frogs were obtained from the European *Xenopus* Resource Centre (University of Portsmouth). Oocytes were isolated and defolliculated as described previously [42] following procedures that have been approved by both UCL's Biological Services Management Group and the UK Home Office (under licences PIL70/23585 and PPL70/06819). Heterologous expression was achieved by injection of cRNA (8–12 ng) into the oocyte cytoplasm. Oocytes were injected in a volume of 32.2 nl using a variable volume Nanoject II microinjector (Drummond Scientific). cRNAs encoding α4 and β2 subunits were injected in a 1∶1 ratio in a total injection volume of 32.2 nl/oocyte. After injection, oocytes were incubated at 18°C in a modified Barth's solution containing: 88 mM NaCl, 1 mM KCl, 0.82 mM MgCl_2_, 0.77 mM CaCl_2_, 2.4 mM NaHCO_3_, and 15 mM Tris, 50 mg/l tetracycline, 50 mg/l penicillin and 50 mg/l streptomycin (pH 7.5 with HCl). Experiments were performed on oocytes 3 to 5 days of injection. Oocytes were placed in a recording chamber (internal diameter, 3 mm), which was continuously perfused with a modified Ringer solution (115 mM NaCl, 2.5 mM KCl, 10 mM HEPES, 1.8 mM BaCl_2_ or CaCl_2_). Oocytes were impaled by two microelectrodes filled with 3 M KCl (0.5–1.5 MΩ) and voltage-clamped at −60 mV using a GeneClamp 500 amplifier (Axon Instruments). Drugs were applied by switching between control and drug-containing solution using a BPS-8 valve control system (ALA Scientific). Agonists were applied for either 5 seconds (acetylcholine) or up to 40 seconds (4BP-TQS) with 2 minute (acetylcholine) or 8 minute (4BP-TQS) intervals between applications. The experiments were performed at room temperature (RT; approx. 21°C). To detect effects of changed temperature on kinetics of nAChRs the modified Ringer solution was warmed/cooled to 37°C or 4°C in water bath or on ice. Agonist solutions were prepared fresh and warmed or cooled for 10–15 min immediately prior to use. In control experiment, it was confirmed that warming agonist solutions, even for 3 hours did not alter agonist potency. To examine effects of temperature, oocytes were continuously perfused with warm/cold modified Ringer solution for 40 sec prior to agonist application. Peak current amplitudes evoked by warm/cold acetylcholine were normalized to the peak amplitudes at RT from the same oocyte. To examine reversibility of any temperature induced changes, oocytes were then perfused with RT Ringer for 5 mins, followed by application of acetylcholine at RT. Peak amplitudes, the rise time and time to decay to half of the maximum amplitude were measured using Clampfit 9.2 (Molecular Devices). Effects of temperature on current amplitude were determined by measuring peak responses, however, results were not significantly different when responses were measure as area above the curve/total charge transfer. Statistical significance was determined using with SigmaStat (Aspire Software International) using non-paired Student's *t*-test. The effects of raising or lowering temperature on agonist-evoked responses were normalized to responses recorded at RT on the same oocyte. All data are reported as mean ± S.E.M.

## References

[1] Lester HA, Dibas MI, Dahan DS, Leite JF, Dougherty DA (2004). Cys-loop receptors: new twists and turns.. Trends Neurosci.

[2] Unwin N (2005). Refined structure of the nicotinic acetylcholine receptor at 4Å resolution.. J Mol Biol.

[3] Millar NS, Gotti C (2009). Diversity of vertebrate nicotinic acetylcholine receptors.. Neuropharmacol.

[4] Arneric SP, Holladay M, Williams M (2007). Neuronal nicotinic receptors: a perspective on two decades of drug discovery research.. Biochem Pharmacol.

[5] D'hoedt D, Bertrand D (2009). Nicotinic acetylcholine receptors: an overview on drug discovery.. Expert Opin Ther Targets.

[6] Haydar SN, Dunlop J (2010). Neuronal nicotinic acetylcholine receptors - targets for the development of drugs to treat cognitive impairment associated with schizophrenia and Alzheimer's disease.. Curr Top Med Chem.

[7] Rollema H, Coe JW, Chambers LK, Hust RS, Stahl SM (2007). Rationale, pharmacology and clinical efficacy of partial agonists of α4β2 nACh receptors for smoking cessation.. Trends Phramacol Sci.

[8] Sine SM, Engel AG (2006). Recent advances in Cys-loop receptor structure and function.. Nature.

[9] Taly A, Corringer P-J, Guedin D, Lestage P, Changeux J-P (2009). Nicotinic receptors: allosteric transitions and therapeutic targets in the nervous system.. Nature Rev Drug Discovery.

[10] Gill JK, Savolainen M, Young GT, Zwart R, Sher E (2011). Agonist activation of α7 nicotinic acetylcholine receptors via an allosteric transmembrane site.. Proc Natl Acad Sci USA.

[11] Young GT, Zwart R, Walker AS, Sher E, Millar NS (2008). Potentiation of α7 nicotinic acetylcholine receptors via an allosteric transmembrane site.. Proc Natl Acad Sci USA.

[12] Collins T, Young GT, Millar NS (2011). Competitive binding at a nicotinic receptor transmembrane site of two α7-selective positive allosteric modulators with differeng effects on agonist-evoked desensitization.. Neuropharmacol.

[13] Couturier S, Bertrand D, Matter JM, Hernandez MC, Bertrand S (1990). A neuronal nicotinic acetylcholine receptor subunit (α7) is developmentally regulated and forms a homo-oligomeric channel blocked by α-BTX.. Neuron.

[14] Revah F, Bertrand D, Galzi JL, Devillers-Thiery A, Mulle C (1991). Mutations in the channel domain alter desensitization of a neuronal nicotinic receptor.. Nature.

[15] Millar NS (2009). A review of experimental techniques used for the heterologous expression of nicotinic acetylcholine receptors.. Biochem Pharmacol.

[16] Bregestovski PD, Chailachjan LM, Dunin-Barkovski VL, Potapova TW, Veprintsev BN (1972). Effect of temperature on the equilibrium endplate potential.. Nature.

[17] Katz B, Miledi R (1972). The statistical nature of the acetycholine potential and its molecular components.. J Physiol.

[18] Kordaš M (1972). An attempt at an analysis of the factors determining the time course of the end-plate current.. J Physiol.

[19] Magleby KL, Stevens CF (1972). A quantitative description of end-plate currents.. J Physiol.

[20] Anderson CR, Stevens CF (1973). Voltage clamp analysis of acetylcholine produced end-plate current fluctuations at frog neuromuscular junction.. J Physiol.

[21] Head SD (1983). Temperature and end-plate currents in rat diaphragm.. J Physiol.

[22] Boyd ND (1987). Two distinct kinetic phases of desensitization of acetylcholine receptors of clonal rat PC12 cells.. J Physiol.

[23] Khmyz V, Maximyuk O, Teslenko V, Verkhratsky A, Krishtal O (2008). P2X_3_ receptor gating near normal body temperature.. Eur J Physiol.

[24] Korinek M, Sedlacek M, Cais O, Dittert I, Vyklicky L (2010). Temperature dependence of *N*-methyl-D-aspartate receptor channels and *N*-methyl-D-aspartate receptor excitatory postsynaptic currents.. Neurosci.

[25] Dickinson R, Lieb WR, Franks NP (1995). The effect of temperature on the interactions between volatile general anaesthetics and a neuronal nicotinic acetylcholine receptor.. Br J Pharmacol.

[26] Sitzia F, Brown JT, Randall AD, Dunlop J (2011). Voltage- and temperature-dependent allosteric modulation of α7 nicotinic receptors by PNU120596.. Front Pharmacol.

[27] Bertrand D, Devillers-Thiery A, Revah F, Galzi JL, Hussy N (1992). Unconventional pharmacology of a neuronal nicotinic receptor mutated in the channel domain.. Proc Natl Acad Sci USA.

[28] Bertrand S, Devillers-Thiéry A, Palma E, Buisson B, Edelstein SJ (1997). Paradoxical allosteric effects of competitive inhibitors on neuronal α7 nicotinic receptor mutants.. NeuroReport.

[29] Paulson HL, Claudio T (1990). Temperature-sensitive expression of all-*Torpedo* and *Torpedo*-rat hybrid AChR in mammalian muscle cells.. J Cell Biol.

[30] Lansdell SJ, Schmitt B, Betz H, Sattelle DB, Millar NS (1997). Temperature-sensitive expression of *Drosophila* neuronal nicotinic acetylcholine receptors.. J Neurochem.

[31] Lansdell SJ, Millar NS (2004). Molecular characterisation of Dα6 and Dα7 nicotinic acetylcholine receptor subunits from *Drosophila*: formation of a high-affinity α-bungarotoxin binding site revealed by expression of subunit chimeras.. J Neurochem.

[32] Lansdell SJ, Collins T, Yabe A, Gee VJ, Gibb AJ (2008). Host-cell specific effects of the nicotinic acetylcholine receptor chaperone RIC-3 revealed by a comparison of human and *Drosophila* RIC-3 homologues.. J Neurochem.

[33] Cooper ST, Harkness PC, Baker ER, Millar NS (1999). Upregulation of cell-surface α4β2 neuronal nicotinic receptors by lower temperature and expression of chimeric subunits.. J Biol Chem.

[34] Nelson ME, Wang F, Kuryatov A, Choi C, Gerzanich V (2001). Fuctional properties of human AChRs expressed by IMR-32 neuroblastoma cells resemble those of α3β4 AChRs expressed in permanently transfected HEK cells.. J Gen Physiol.

[35] Williams DK, Wang J, Papke RL (2011). Positive allosteric modulators as an approach to nicotinic acetylcholine receptor-targeted therapeutics: advantages and limitations.. Biochem Pharmacol.

[36] Sine SM, Steinbach JH (1984). Activation of a nicotinic acetylcholine receptor.. Biophys J.

[37] Quartararo N, Barry PH (1988). Ion permeation through single ACh-activated channels in denervated adult toad sartorius skeletal muscle fibres: effect of temperature.. Pflugers Arch.

[38] Dilger JP, Brett RS, Poppers DM, Liu Y (1991). The temperature dependence of some kinetic and conductance properties of acetylcholine receptor channels.. Biochim Biophys Acta.

[39] Zanello LP, Aztiria E, Antollini S, Barrantes FJ (1996). Nicotinic acetylcholine receptor channels are influenced by the physical state of their membrane environment.. Biophys J.

[40] Gupta S, Auerbach A (2011). Temperature dependence of acetylcholine receptor channels activated by different agonists.. Biophys J.

[41] Broadbent S, Groot-Kormelink PJ, Krashia PA, Harkness PC, Millar NS (2006). Incorporation of the β3 subunit has a dominant-negative effect on the function of recombinant central-type neuronal nicotinic receptors.. Mol Pharmacol.

[42] Young GT, Broad LM, Zwart R, Astles PC, Bodkin M (2007). Species selectivity of a nicotinic acetylcholine receptor agonist is conferred by two adjacent extracellular β4 amino acids that are implicated in the coupling of binding to channel gating.. Mol Pharmacol.

